# Chemical Composition and Biological Activity of *Commelina erecta*: An Edible Wild Plant Consumed in Brazil

**DOI:** 10.3390/foods12010192

**Published:** 2023-01-01

**Authors:** Lucas Vinicius Cavichi, Ângela Liberal, Maria Inês Dias, Filipa Mandim, José Pinela, Marina Kostić, Marina Soković, Daneysa Lahis Kalschne, Ângela Fernandes, Cristiane Canan, Lillian Barros, Joana S. Amaral

**Affiliations:** 1 Centro de Investigação de Montanha (CIMO), Instituto Politécnico de Bragança, Campus de Santa Apolónia, 5300-253 Bragança, Portugal; 2 Laboratório Associado para a Sustentabilidade e Tecnologia em Regiões de Montanha (SusTEC), Instituto Politécnico de Bragança, 5300-253 Bragança, Portugal; 3 Department of Food Sciences, Federal Technological University of Paraná, Medianeira 85884-000, Brazil; 4 Institute for Biological Research “Siniša Stanković”-National Institute of Republic of Serbia, University of Belgrade, 11000 Belgrade, Serbia

**Keywords:** *Commelina erecta*, bioactive properties, chemical profile, nutritional, wild edible plants

## Abstract

In recent years, the interest in products of natural origin has boosted the exploitation and use of plants as food and sources of bioactive compounds, especially wild plants widely used in different cultures for several purposes. *Commelina erecta* is a wild edible plant (WEP) traditionally used as food and medicine, about which few studies exist. Thus, this study aimed at enhancing the knowledge about its nutritional, chemical and bioactive profile, considering different plant parts and development stages, in order to increase its inclusion in the diet of South American communities. The nutritional profile was found to be similar to other WEP frequently consumed in Brazil. Thirteen phenolic compounds (HPLC-DAD-ESI/MS) were tentatively identified, with apigenin, luteolin and quercetin derivatives being the most abundant. Fructose and oxalic acid were the major sugar and organic acid, respectively, in the aerial parts of *C. erecta*, and four isoforms of tocopherols were also identified. Regarding the plant’s antioxidant activity, the EC_50_ values varied between 18.4 and 1060 µg/mL in the inhibition of lipid peroxidation assay (TBARS) and between 53 and 115 µg/mL in the oxidative haemolysis inhibition (OxHLIA) assay. The hydroethanolic extract obtained from stems at the flowering stage also presented anti-inflammatory activity. In general, all the extracts evidenced promising antimicrobial activity. Altogether, these results reinforce the traditional use of this plant species as food and medicine to support the diet of needier populations and also promote food sovereignty and sustainability.

## 1. Introduction

In recent years, there has been a growing interest in functional foods and medicines obtained from natural sources. Plants are matrices naturally rich in bioactive compounds, and wild plants, in particular, have long played a significant role in different cultures [1]. Besides their importance in traditional medicine, native wild plants are a relevant source of nutrients in times of scarcity, such as during periods of war, and have long been used to complement and diversify the diet, especially in rural areas. The recurrence of its use is mainly related to cultural factors, where knowledge is passed on to the newest generations, and to economic reasons due to low incomes and the absence of financial support [2,3]. Nowadays, several wild plants are frequently perceived as weeds that should be eliminated as they reproduce quickly in diverse environments, grow together undesirably and compete with crops. However, some of them are still used as foods by rural communities and have a high potential as they are edible and of great ecological importance. Therefore, to preserve the knowledge transmitted among generations and obtain new data and information on these still underexplored genetic resources, the scientific community has been performing various studies including ethnobotanical surveys, the chemical characterization of the diversity of wild plants, and the study of their biological activities [4]. Allied to scientific studies, gastronomic trends are also emerging to add value to wild regional plants in modern dishes [5].

*Commelina erecta* L. is a wild edible plant belonging to the Comelinaceae family native to tropical America and widespread in Brazil, where it is known as trapoeraba-azul or erva de Santa-Luzia. Its aerial parts, namely stems and leaves, are used as food either by cooking, canning or raw in salads [6,7,8,9,10]. Besides being used as an unconventional food plant, its use for phytotherapeutic purposes as a traditional medicine is also reported, mainly in the treatment of sore throat, eye infections and inflammations, wound healing and dermatological problems, but also in women’s infertility, diabetes, rheumatism, hypertension and diarrhoea [11,12,13,14,15]. Although several studies have already investigated other species of the same genus, namely *Commelina benghalensis* [1,8,16,17,18,19], *Commelina diffusa* [20,21] and *Commelina nudiflora* [22,23,24], there is still scarce information regarding *C. erecta*.

Recently, Otsuka et al. [25] reported the presence of rutin and caffeic acid in the ethanolic and aqueous extracts of *C. erecta* leaves, and Bezerra et al. [26] identified the presence of luteolin, isoquercitrin, quercitrin, shikimic acid, saccharinic acid lactone and three sterols in the ethyl acetate and ethanolic extracts of the stems. Moreover, the antioxidant activity [25,26] and antimicrobial activity by the agar-diffusion method [27] were evaluated for this species. Although being used by different communities in South America, the utilization of this species is based only on empirical knowledge because there is a scarcity of data on its nutritional and chemical composition as well as on its bioactive properties. Therefore, this work aims at contributing to validate this empirical knowledge by conducting a thorough characterization of its nutritional profile and chemical composition, complemented by an evaluation of the bioactivity of the aqueous and hydroethanolic extracts against oxidation, inflammation, and the growth of bacteria and fungi. This evaluation also investigated the influence of harvest time with samples collected during and after the flowering stage.

## 2. Materials and Methods

### 2.1. Samples

The edible parts of *C. erecta* (leaves and stems) were harvested in 2020, during the flowering and post-flowering period, in the western region of Paraná, Brazil. The samples were collected, cleaned and lyophilized (*L101* LIOTOP, Liobras, São Paulo, Brazil). They were then reduced to a fine dried powder (20 mesh), mixed to obtain homogenate samples, and stored at −20 °C until analysis.

### 2.2. Preparation of Extracts

Hydroethanolic and decoction extracts were prepared using 2.5 g and 1 g of lyophilized sample, respectively. The former was obtained using an ethanol:water solution (80:20, *v/v*) under magnetic agitation for 1 h. The residue obtained after filtration (filter paper Whatman N.° 4) was reextracted. The combined filtrates were then rotoevaporated (rotary evaporator Büchi R-210, Flawil, Switzerland) under pressure at 40 °C and 100 mbar and subsequently freeze-dried. For the decoctions, 100 mL of boiling distilled water was added to the sample. The mixture was boiled for 5 min on a heating plate (VELP Scientifica, Usmate, Italy), filtered (filter paper Whatman N.° 4), frozen and freeze-dried to obtain an extract.

### 2.3. Chemical Parameters

#### 2.3.1. Nutritional Value

Moisture was determined in the fresh samples by weighing the lyophilized samples, and the lipid, ash, protein and carbohydrate contents were determined in the lyophilized samples according to the methodologies proposed by AOAC [28]. For the calculation of crude protein, a factor of N × 6.25 was used. Total carbohydrate was calculated by difference, and the energy (kcal/100 g of fresh weight (fw)) was calculated according to the Atwater system using the formula: 4 kcal/g × (g proteins + g carbohydrates) + 9 kcal/g × (g lipids).

#### 2.3.2. Free Sugars

Soluble free sugars were determined by high-performance liquid chromatography with a refractive index detector (HPLC-RI; Knauer, Smartline system 1000, Berlin, Germany) using 1 g of lyophilized sample following a previously described methodology [29]. The identification was carried out by comparison with standards and quantification used melezitose as the internal standard. The results were recorded and processed using Clarity 2.4 software (DataApex, Prague, Czech Republic) and expressed as g per 100 g of fw.

#### 2.3.3. Organic Acids

The organic acids were extracted from 1.5 g of the lyophilized plant using a previously described methodology [29] and analyzed using an ultrafast liquid chromatography system coupled to a diode array detector (UPLC-DAD; Shimadzu series 20 A UFLC, Shimadzu Corporation, Kyoto, Japan). Standards of oxalic, quinic, malic, ascorbic, citric and fumaric acids (Sigma-Aldrich, St. Louis, MO, USA) were used for the identification of compounds and for obtaining the calibration curves used for quantification (ascorbic acid was quantified at 245 nm and the remaining acids at 215 nm). The results were expressed as mg per 100 g of fw.

#### 2.3.4. Fatty Acids

Fatty acids were determined in the lipid fraction obtained from Soxhlet extraction after being trans-esterified to fatty acid methyl esters (FAME) following a methodology previously described [29]. The FAME were determined by gas-liquid chromatography with flame ionization detection, using a YOUNG IN Chromass6500 GC System apparatus equipped with a *split/splitless* injector, a flame ionization detector (FID) and a Zebron-Fame column. A mixture of 37 FAME standards (47885-U; Sigma-Aldrich, St. Louis, MO, USA) was used for the identification of compounds. The Clarity DataApex 4.0 Software (DataApex, Prague, Czech Republic) was utilized for data handing. The results were expressed as the relative percentage (%) of each detected fatty acid.

#### 2.3.5. Tocopherols

Tocopherols were determined in approximately 500 mg of the lyophilized plant material using a high-performance liquid chromatography system coupled to a fluorescence detector (HPLC-FL; Knauer, Smartline system 1000, Berlin, Germany) as previously described by [29]. The quantification of α-, β-, γ- and δ-tocopherols was performed based on the calibration curves constructed using authentic standards (Sigma, St. Louis, MO, USA) and tocol (Matreya, Pleasant Gap, PA, USA) as an internal standard. The results were recorded and processed using Clarity 2.4 software (DataApex, Prague, Czech Republic) and expressed in mg per 100 g fw.

#### 2.3.6. Phenolic Compounds

Phenolic compounds were analyzed by high performance liquid chromatography coupled to a diode detector and mass spectrometer (HPLC-DAD-ESI-MS/MS) operating under the conditions proposed by Bessada et al. [30]. For that purpose, the hydroethanolic and decoction extracts were redissolved in methanol/water (80:20, *v/v*) to a final concentration of 5 mg/mL and filtered using a 0.22 μm disposable filter disc. The identification of compounds was achieved by comparing the obtained retention times, UV-VIS and mass spectra with those of the available standards. When standards were not available, the compounds were tentatively identified based on the fragmentation pattern and data from the literature. The identified compounds were quantified using the calibration curves in the range of 200–5 μg/mL obtained from caffeic acid, ferulic acid and quercetin-3-*O*-glycoside standards (Extrasynthese, Genay, France). The results were expressed in mg per g of extract.

### 2.4. Bioactive Properties

#### 2.4.1. Evaluation of In Vitro Antioxidant Properties

The antioxidant activity of the extracts was determined by two in vitro assays, namely, the inhibition of lipid peroxidation by evaluating the thiobarbituric acid reactive substances (TBARS) formation using porcine brain homogenates, and the inhibition of oxidative haemolysis (OxHLIA) in sheep’s blood erythrocytes, as described by [31]. The EC_50_ values (sample concentration providing 50% of antioxidant activity or 50% haemolysis inhibition for ∆*ts* of 60 and 120 min) were used to express the results. Trolox (Sigma-Aldrich, St. Louis, MO, USA) was used as a positive control.

#### 2.4.2. Anti-Inflammatory Activity

The anti-inflammatory activity of the extracts (400 to 6.25 μg/mL) was evaluated based on nitric oxide (NO) production in a RAW 264.7 murine macrophage cell line (ECACC 91062702) due to lipopolysaccharide (LPS, 1 mg/mL in DMEM; Sigma-Aldrich, Saint Louis, MO, USA) stimulation. For that purpose, the Griess Reagent System kit (Promega, Madison, WI, USA) was used as described in previously published protocols [32]. Dexamethasone (50 mM) (Sigma-Aldrich, Saint Louis, MO, USA) and samples without the addition of LPS were used as positive and negative controls, respectively. The NO generated was monitored at 540 nm (ELX800 Biotek microplate reader; Bio-Tek Instruments Inc., Winooski, VT, USA). The extract concentrations that trigger 50% of NO production inhibition were expressed as EC_50_ values (μg/mL).

#### 2.4.3. Antimicrobial Activity

Three Gram-positive bacteria (*Staphylococcus aureus* (ATCC 11632), *Bacillus cereus* (food isolate) and *Listeria monocytogenes* (NCTC 7973)) and three Gram-negative bacteria (*Escherichia coli* (ATCC 25922), *Salmonella enterica* subsp*. enterica* serovar Typhimurium(ATCC 13311) and *Enterobacter cloacae* (clinical isolate)) were selected to test the antibacterial activity of the extracts.

For antifungal activity, six micromycetes were used, namely, *Aspergillus fumigatus* (clinical isolate), *Aspergillus Niger* (ATCC 6275), *Aspergillus ochraceus* (ATCC 12066), *Penicillium funiculosum* (ATCC 36839), *Penicillium verrucosum var. cyclopium* (food isolate) and *Trichoderma viride* (IAM 5061).

The microdilution method to determine the minimum inhibitory, bactericidal and fungicidal concentrations (MICs, MBCs and MFCs, expressed in mg per mL) was performed as previously described by the authors [33]. The food preservatives sodium sulphite (E221) and potassium metabisulphite (E224) (Sigma-Aldrich, St. Louis, MO, USA) were used as positive controls.

### 2.5. Statistical Analysis

All assays were performed in triplicate (*n = 3*) and the values were expressed as mean ± standard deviation (SD). Statistical tests were performed with a significance level of 5%, using the statistical software SPSS (IBM SPSS Statistics for Windows, Version 23.0; IBM Corp., Armonk, NY, USA). The differences between the samples were evaluated by unidirectional analysis of variance (ANOVA) and the means were compared according to the Tukey’s HSD test (*p* = 0.05).

## 3. Results and Discussion

### 3.1. Chemical and Nutritional Characterization of C. erecta Aerial Parts

In this work, the leaves and stems of *C. erecta* were studied in different collection periods, namely at the flowering stage and post-flowering, since it is known that several factors and different stimuli, such as seasonal and environmental stress, climate, soil, and harvest period, among others, can influence the chemical composition of plants [34]. Table 1 shows the results obtained for the nutritional composition of the evaluated aerial parts of *C. erecta*.

A lower moisture content was found for the post-flowering samples, corresponding to a higher variation in the macronutrient concentration related to the harvest period. This was particularly noticeable in the case of lipids that showed an increase of 14× and 23× from the flowering to the post-flowering stage in the leaf and stem, respectively. The protein content was higher in the leaves compared with the stems for both collecting periods. Carbohydrates were the most abundant macronutrients in all samples, with higher values being obtained also in the post-flowering stage. Overall, the samples harvested after flowering presented a higher energy content despite all being low in total calories content. Comparing the obtained results with those reported for other wild edible plants popularly consumed in Brazil, such as *Eruca vesicaria* (Arugula), *Pereskia aculeata* (Ora-pro-nóbis), *Portulaca oleracea* (Beldroega), and *Spilanthes oleracea* L. (Jambu) that are widely used in typical Amazonian dishes [35,36,37], the macronutrient values of trapoeraba-azul (*C. erecta*) are generally identical or higher, with the exception of the protein content that was slightly higher in these species (from 2.58 to 3.39 g/100 g fw) (Supplementary material, Table S1). Thus, the insertion of this species as part of a varied diet is interesting given the similarity with other unconventional food plants that have higher levels of use for food consumption.

As shown in Table 1, one disaccharide and two monosaccharides were identified, namely sucrose, glucose and fructose. Of these, fructose was present in all evaluated edible parts, differing from sucrose that was only detected in the post-flowering leaf in residual concentrations. We also observed an increase in the concentration of total free sugars in the post-flowering period, which may be related to the decrease in the need for energy used by the plant to form the flower [38]. Among the studied samples, the stem after flowering presented the highest total free sugar content.

Regarding organic acids, four compounds were found: oxalic, malic, shikimic and ascorbic acids, as shown in Table 1. Overall, the aerial part with the highest concentration of organic acids was the post-flowering stem. This sample also had the highest content of oxalic acid, which can promote kidney stones and interfere in calcium absorption. Although the stems were richer in this acid compared with the leaves, the contents found were still lower than those reported for widely consumed vegetables such as *Spinacia oleracea* (spinach), root *Apium graveolens* (celery), and *Petroselinum crispum* (parsley), as well as for some other wild edible plants such as *Raphanus raphanistrum* L. (wild radish, 706 mg/100 g fw) [4,39]. The stems also presented a considerable content of shikimic acid, which is in agreement with previous studies that identified this compound in *C. erecta* stems [26]. Malic acid, which is associated with antioxidant potential due to its chelating power [30], was the second most abundant organic acid in both the stem and leaf at the flowering stage. On the other hand, the antioxidant ascorbic acid was not detected in the flowering leaf, with the content of this organic acid increasing when samples were collected in the post-flowering period (Table 1).

The results obtained for the analysis of the fatty acid profile are shown in Table 2. In total, 18 fatty acids were identified, with polyunsaturated fatty acids (PUFA) being the major group, followed by saturated fatty acids (SFA) and, in smaller quantities, monounsaturated fatty acids (MUFA). There were significant differences among the samples with a considerable variation in PUFA (ranging from 44.8% to 62.7%) and SFA (26.4% to 44.4%).

At both stages of plant development, PUFA content was notably higher in the leaf compared with the stem mainly due to the α-linolenic acid content (approximately 43% vs. 18%, respectively), although the stems were slightly richer in linoleic acid (approximately 20% vs. 27% in the leaves and stems, respectively). The opposite occurred for the SFA, with stems being much richer in palmitic acid than the leaves (approximately 27% vs. 19% mean value). Therefore, it can be concluded that the fatty acid profile of the leaves is more beneficial for health since the consumption of omega-3 fatty acids has been associated with a lower risk of developing cardiovascular diseases [40]. Moreover, the leaves also presented a higher total tocopherol content than that found in the stems, which may be related to the higher PUFA content of these samples. The leaf at the flowering stage was the sample with the highest total tocopherols content and the only one that presented the four tocopherol’s isoforms (α, β, γ, δ).

The analysis of phenolic compounds in the leaves and stems of *C. erecta* was carried out by HPLC-DAD-ESI/MS/MS. Table 3 presents the data regarding chromatographic responses and tentative identification of the phenolic compounds found in the studied samples, and Table 4 presents the corresponding quantification. Overall, thirteen compounds were identified, namely ten *C*-glycosylated derivatives of apigenin, luteolin, and daidzin (puerarin) and three *O*-glycosylated derivatives of quercetin.

Peaks **1** and **2** presented the same deprotonated ion [M-H]^−^ at *m/z* 593; however, they belong to different categories: peak **1** can be classified as a flavonoid-mono-*C*-glycoside-*O*-glycoside whereas peak **2** is a flavonoid-di-*C*-glycoside [41]. Peak **2**, besides the characteristic UV spectra, also presented characteristic MS^2^ fragments of apigenin aglycone (269 u) at *m/z* 383 (+113 u) and 353 (+83 u). A major fragment at *m/z* 473 (−120 u) that coupled with the mass of the molecule indicates the presence of two hexosyl units, being tentatively identified as apigenin-6,8-di-*C*-hexoside. On the other hand, in peak **1**, it was possible to observe characteristic MS^2^ fragments of the mono-*C*-glycosylation of the molecule at *m/z* 311 that correspond to the sum of the apigenin aglycone (270 MW) with 41 u, and there were also fragments at *m/z* 503 (−90 u) and 473 (−120 u) corresponding to the hexosyl unit linked to *C*-linkage. Moreover, we also observed a base peak at *m/z* 431 (- 162 u) that corresponds to the *O*-hexosylation over a phenolic hydroxyl [41], and therefore, this peak was tentatively identified as apigenin-7-*O*-hexosyl-8-*C*-hexoside. Peak **9**, also a di-*C*-glycoside, presented the characteristic fragments at *m/z* 383 (−90 u) and 353 (−60 u) of the apigenin aglycone that also corresponds to the cleavage of the glycosidic bond of the *C*-8-pentose. Furthermore, with a deprotonated ion [M-H]^−^ at *m/z* 533, a base peak at *m/z* 443 (−90 u), and another MS fragment at *m/z* 473 (−60 u), it can also be inferred that another *C*-pentosyl moiety is linked to the molecule. Based on these data and information from the literature [42], peak **9** was tentatively identified as apigenin-6,8-di-*C*-pentoside.

Peaks **5** and **6** presented the same deprotonated ion [M-H]^−^ at *m/z* 563, characteristic fragments at *m/z* 383 and 353 (apigenin aglycone), a major ion fragment at *m/z* 443 (loss of 120 u, hexosyl unit), and one fragment at *m/z* 503 (loss of 60 u, pentosyl unit). The differentiation between the two peaks was performed taking into account the abundance of the −60 unit loss (*m/z* 503) since, according to Ferreres et al. [41], a high abundance indicates that the pentosyl unit is located in the 6-*C* position as the sugar moiety suffers a preferential fragmentation compared with the one located in position 8-*C*. In this manner, peaks **5** and **6** were tentatively identified as apigenin 6-*C*-hexosyl-8-*C*-pentoside (12% abundance of fragment at *m/z* 503) and apigenin 6-*C*-pentosyl-8-*C*-hexoside (71% abundance of fragment at *m/z* 503), respectively.

Peaks **3**/**4** and **7**/**8** were tentatively identified following the description previously reported by Figueirinha et al. [43] and Ferreres et al. [41]. Peaks **3**/**4** presented a deprotonated ion [M-H]^−^ at *m/z* 579 and high abundance of MS^2^ fragments at *m/z* 489 (−90 u) and 459 (−120 u), which suggest the 6-*C* position of the hexosyl unit, and low abundance of the fragments at *m/z* 519 (−60 u), 399 (−120–60 u), and 369 (−120–90 u) corresponding to the *C*-pentosyl unit. The presence of *m/z* 399 (285 u + 83 u) and 369 (285 u + 113 u) is in accordance with the presence of a tetrahydroxylated flavone (luteolin aglycone, *m/z* 285), being tentatively identified as luteolin-6-*C*-hexosyl-8-*C*-pentoside derivative I (peak **3**) and II (peak **4**).

Peaks **7**/**8**, similar to peaks **3**/**4**, also presented the MS^2^ fragments at *m/z* 399 and 369 characteristic of luteolin aglycone. However, the deprotonated molecule [M-H]^−^ at *m/z* 549 and following fragments at *m/z* 531 (−18 u), 489 (−60 u), 459 (−90 u), and 429 (−120 u), which are consistent with two pentosyl units, lead to the tentative identification of the peaks as luteolin-6-*C*-pentosyl-8-*C*-pentoside derivative I (peak **7**) and II (peak **8**) [43].

Peak **10**, tentatively identified as hydroxyl-puerarin, was included in the *C*-glycosylated derivatives, since it corresponds to the *C*-glycosylated form of the isoflavone daidzin. This peak presented a deprotonated molecule at at *m/z* 431 and a prominent MS^2^ fragment at *m/z* 311 that corresponded to the loss of 120 u, the characteristic mass loss of *C*-glycosylated molecules. The remaining mass fragmentation and UV spectra responses were used for the tentative identification of this compound, which was in accordance with previously reported information [44,45].

Finally, for the *O*-glycosylated derivatives, peak **11** ([M-H]^−^ at *m/z* 463) was identified as quercetin-3-*O*-hexoside based on the comparison of the retention time and UV-vis spectra of the available standard compound. Peak **12** presented a deprotonated molecule [M-H]^−^ at *m/z* 549 and subsequent MS^2^ fragments at *m/z* 505, 463, and 301 (quercetin aglycone) that correspond to the loss of a malonyl (44 u + 42 u) and hexosyl moieties (162 u) and was tentatively identified as quercetin-*O*-malonyl-hexoside. Peak **13** ([M-H]^−^ at *m/z* 447) presented a simpler mass response with only one MS^2^ fragment at *m/z* 301 (146 u) and was tentatively identified as quercetin-*O*-deoxyhexoside.

In all the studied samples, the presence of apigenin derivatives was verified, whereas the luteolin derivatives were detected only in the stem of *C. erecta.* The *C*-glycosylated derivatives represent around 77% of the total compounds found in the samples of *C. erecta*, mainly due to the presence of apigenin derivatives followed by luteolin.

This is in good agreement with the results of Martínez and Swain [46] who reported a predominance of flavone *C*-glycosides in different species of the Commelinaceae family, including *C. erecta* which also presented flavonol *O*-glycosides derived from quercetin. Although the present study revealed the presence of flavonol *O*-glycosides, namely quercetin derivatives, except for the hydroethanolic extract prepared from the stem at the flowering stage that presented higher amounts, only traces of these compounds were detected in some of the other extracts. The presence of quercetin glycosides, more specifically quercetin-3-*O*-hexoside and quercetin-*O*-deoxyhexoside, is in accordance with the data previously reported by Bezerra et al. [26] who observed the presence of quercitrinand isoquercitrin in the stem of *C. erecta*. In the same study, using mass spectrometry and NMR, the authors also identified the presence of luteoline aglycone, which was identified in the present study in the form of heterosidic derivatives. However, in most of the analyzed samples, only traces of these compounds were found, being possible to quantify them only in the hydroethanolic extract obtained from the stem at the flowering stage. A fourth compound, namely rutin, has also been identified in previous studies of *C. erecta* leaves [25]. In the works of both Bezerra et al. [26] and Otsuka et al. [25], these compounds were only identified with no quantification being performed; thus, no comparison of values can be carried out. Among the identified phenolic compounds (Table 3), it is noteworthy that several apigenin derivatives are reported for the first time in this species. Apigenin was the most frequent aglycone in the evaluated edible parts, with all the samples presenting at least one of its derivatives. Recent studies have identified apigenin as a phenolic compound with great nutraceutical potential due to its ability to scavenge free radicals and is thus associated with neuroprotective effects, and anticancer and anti-inflammatory activity [47,48]. Comparing the values obtained for the two harvesting periods, it can be noticed that higher content of phenolic compounds was observed for both plant parts during the flowering stage, which could be related to the higher protection required at this stage. Moreover, the composition between stems and leaves was observed to be different. Curiously, higher amounts of total phenolics were obtained in the hydroethanolic extract for the stems and aqueous extract for the leaves.

### 3.2. Bioactive Properties of C. erecta Extracts

The results of the antioxidant activity assays obtained for the two extracts (hydroethanolic and aqueous) prepared with the different plant parts are presented in Table 5. The best results in the OxHLIA assay were observed for the aqueous extract of the leaf at flowering stage, whereas the aqueous extract of the stem after flowering stage performed better in the TBARS assay. Curiously, this last extract presented a low performance in OxHLIA assay similar to the hydroethanolic extract obtained from the same part of the plant. This lower antioxidant activity could be related to the lower content of tocopherols detected in the stem after flowering. Despite not having better antioxidant results, the hydroethanolic extract prepared from the stem at the flowering stage consistently presented good antioxidant properties in both assays (the EC_50_ was approximately 4 times higher than the synthetic antioxidant used as positive control in OxHLIA assay and 14 times higher in TBARS assay), which could be related to its higher content in phenolic compounds. The antioxidant activity of *C. erecta* stems, namely its hexane, ethyl acetate and ethanolic extracts, has been previously evaluated by Bezerra et al. [26], who reported a weak activity for all extracts in both DPPH and ABTS assays. A similar result was reported by Otsuka et al. [25], who also described a weak antioxidant activity for the aqueous (infusion) and ethanolic extracts prepared from *C. erecta* dried leaves, particularly in the DPPH assay where the aqueous and ethanolic extracts presented inhibition levels of only 5.4% and 4.6%, respectively. Although different assays have been used to assess the antioxidant properties of the samples, in general, better results were obtained in the present work. This could be related to the higher quantity and diversity of phenolic compounds identified in the herein studied samples. However, a quantitative comparison with the previous works is not feasible since the authors evaluated only the total phenolics content using the *Folin*–*Ciocalteu* reagent, with no quantification of the individual compounds identified by HPLC-MS being performed.

Considering the phytopharmaceutical use of the leaves, flowers and stems of *C. erecta* in traditional medicine, the prepared extracts were also evaluated for their anti-inflammatory properties. As can be observed in Table 5, at the tested concentrations, only the hydroethanolic extract obtained from the stems at the flowering stage evidenced anti-inflammatory activity, which is most probably related to its higher diversity and quantities of phenolic compounds.

The results of the antimicrobial activity are shown in the Table 6. The hydroethanolic and aqueous extracts were tested against a panel comprising six bacteria and six fungi, which were selected due to a higher incidence and risk to human health when associated with food-borne diseases.

All the extracts showed both bacteriostatic and bactericidal activity, with MIC and MBC values ranging from 0.25 to 4 mg/mL and 0.5 to 4 mg/mL, respectively. For some bacteria, better results were obtained than with the two food additives used as positive controls (sodium sulphite and potassium metabisulphite). In particular, compared with E211, lower MICs were obtained for all extracts against *S. aureus*, and lower or identical values against *L. monocytogenes*, *E. coli* and *E. cloacae*. Regarding *B. cereus*, all the extracts performed better than E224 with lower MIC and MBC values being obtained. Except for the aqueous extract prepared from the leaf at the flowering stage, identical MIC values against *S.* Thyphimurium were obtained for E211 and the remaining extracts. Overall, the hydroethanolic extract prepared from the leaf at the flowering stage showed better results, which is demonstrated by its lower MIC and MBC values against the majority of the bacteria assayed, particularly against *B. cereus* (MIC 0.25 mg/mL and MBC 0.5 mg/mL), *L. monocytogenes* and *E. cloacae* (MIC 0.5 mg/mL and MBC 1 mg/mL). Nevertheless, this extract was not richer in phenolic compounds, which suggests that other components not evaluated in this work may contribute to the global antimicrobial activity of the extract.

In general, the results obtained in this work are in agreement with those reported by Paz et al. [27] who reported that an aqueous extract of *C. erecta* leaf was effective against the growth of *E. coli* in tests using diffusion methodology on agar (diameter of problem inhibition zone, dr > 0.7). However, the same study suggested the inefficacy of the extract against *S. aureus*, which diverges from the results of the present study that demonstrated both inhibitory and bactericidal effects. This divergence may be related to seasonal and stress factors suffered by the plant since these factors influence the formation of secondary compounds in plants.

As for antifungal activity, the hydroethanolic extracts generally proved to be more effective against the evaluated *Aspergillus* species, whereas the aqueous extracts showed better results against *T. viride* and *Penicillium* species (Table 6). In addition, it should be noted that most of the extracts showed better or identical results compared with the food preservatives used as positive controls.

## 4. Conclusions

Nowadays, the topic of the valorization of wild edible plants is arousing a growing interest in different regions of the globe. Thus, the main aim of this study was to increase the knowledge about the nutritional and chemical composition of *C. erecta* and contribute to consolidating the empirical knowledge of its use in diets in South American countries. Moreover, since it is known that the collecting period can affect the plant’s composition, the aerial edible parts, namely the leaves and stems, were collected at both the plant’s flowering and post-flowering stages. To the best of our knowledge, the nutritional composition in terms of major compounds, fatty acids, tocopherols (vitamin E) and organic acids including ascorbic (vitamin C) is described here for the first time. Despite lipids being present in low amounts, this species revealed a very interesting profile with PUFA being the main fatty acids group. Moreover, a total of 13 phenolic compounds were tentatively identified with most being described in this species for the first time. The compounds were mainly flavone C-glucosides, highlighting the presence of several apigenin derivatives.

As for the plant’s bioactive potential, all the edible parts evidenced antioxidant activity in both the TBARS and OxHLIA assays and presented higher values compared with the positive control (Trolox). The same was observed for the hydroethanolic extracts obtained from the stems at the flowering stage that presented anti-inflammatory activity, although the activity was almost 25× lower than the corticosteroid dexamethasone. Moreover, in general, all the extracts showed very good antimicrobial and antifungal activity, and in some cases, better results for inhibitory and bactericidal activity were obtained than for the two food additives used as positive controls.

Altogether the results obtained in this study support the traditional use of this plant species as a food to supplement the diet of low-income populations and simultaneously contribute to food sovereignty and sustainability.

## Figures and Tables

**Table 1:** Nutritional and energetic value, free sugars and organic acids of the studied *C. erecta* edible parts (mean ± SD, *n* = 3).

	Flowering Stem	Stem after Flowering	Flowering Leaf	Leaf after Flowering
**Nutritional value** (g/100 g fw ^1^)				
Moisture	94 ± 6 ^a^	90.7 ± 9 ^b^	94 ± 6 ^a^	89 ± 11 ^c^
Lipids	0.08 ± 0.01 ^d^	1.86 ± 0.06 ^b^	0.14 ± 0.01 ^c^	2.03 ± 0.04 ^a^
Protein	0.53 ± 0.03 ^d^	0.97 ± 0.01 ^c^	1.64 ± 0.07 ^b^	1.99 ± 0.05 ^a^
Ash	0.73 ± 0.01 ^d^	1.02 ± 0.02 ^c^	0.92 ± 0.02 ^b^	1.39 ± 0.02 ^a^
Carbohydrates	4.55 ± 0.01 ^b^	5.5 ± 0.3 ^a^	3.50 ± 0.07 ^c^	5.9 ± 0.9 ^a^
Energy (kcal/100 g fw)	21.09 ± 0.03 ^c^	42.6 ± 0.7 ^b^	21.82 ± 0.04 ^d^	50 ± 3 ^a^
**Free sugars** (g/100 g fw)				
Fructose	0.079 ± 0.001 ^d^	0.540 ± 0.002 ^a^	0.212 ± 0.003 ^c^	0.311 ± 0.004 ^b^
Sucrose	tr ^2^	tr	0.014 ± 0.002	tr
Total free sugars	0.079 ± 0.001 ^c^	0.540 ± 0.002 ^a^	0.230 ± 0.004 ^d^	0.311 ± 0.004 ^b^
**Organic acids** (mg/100 g fw)				
Oxalic	256.8 ± 0.1 ^c^	507 ± 1 ^a^	208 ± 1 ^d^	383 ± 1 ^b^
Malic	160 ± 2 ^a^	84 ± 1 ^b^	68 ± 1 ^c^	39 ± 1 ^d^
Shikimic	41.4 ± 0.1 ^c^	98.01 ± 0.01 ^b^	9.6 ± 0.1 ^d^	98.1 ± 0.2 ^a^
Ascorbic	2.18 ± 0.09 ^c^	9.5 ± 0.8 ^a^	nd	7.1 ± 0.1 ^b^
Total organic acids	460 ± 2 ^c^	699 ± 1 ^a^	285 ± 2 ^d^	527 ± 2 ^b^

^1^ fw—fresh weigh; tr—traces ^2^. Statistically significant differences (*p* < 0.05) between samples were assessed by a one-way ANOVA, using Tukey’s significant difference (HSD) and are indicated by different letters.

**Table 2:** Composition of fatty acids and of tocopherols of the studied *C. erecta* edible parts (mean ± SD, *n* = 3).

	Flowering Stem	Stem after Flowering	Flowering Leaf	Leaf after Flowering
**Fatty acids (relative %)**				
C6:0	0.36 ± 0.01	nd	nd	nd
C8:0	0.11 ± 0.01	nd	nd	nd
C11:0	0.12 ± 0.01	nd	nd	nd
C12:0	nd	nd	nd	nd
C13:0	0.48 ± 0.01	nd	0.76± 0.04	1.08 ± 0.01
C14:0	0.45 ± 0.01	nd	0.33 ± 0.01	nd
C15:0	0.31 ± 0.02	nd	nd	nd
C16:0	23.31 ± 0.01	30.51 ± 0.05	21.9 ± 0.2	16.60 ± 0.07
C16:1	2.08 ± 0.04	0.94 ± 0.01	2.8 ± 0.1	nd
C17:0	0.99 ± 0.01	0.65 ± 0.01	nd	nd
C18:0	6.41 ± 0.08	4.60 ± 0.05	4.28 ± 0.02	4.91 ± 0.05
C18:1n9c	15.1 ± 0.1	8.11 ± 0.07	4.22 ± 0.04	10.9 ± 0.1
C18:2n6c	26.95 ± 0.04	28.5 ± 0.1	19.24 ± 0.02	20.03 ± 0.02
C18:3n3	17.9 ± 0.2	18.17 ± 0.05	43.6 ± 0.3	42.74 ± 0.08
C20:0	0.95 ± 0.01	nd	1.72 ± 0.08	1.57 ± 0.08
C22:0	1.68 ± 0.04	2.70 ± 0.09	nd	nd
C23:0	1.11 ± 0.03	2.48 ± 0.01	nd	nd
C24:0	1.82 ± 0.02	3.50 ± 0.06	1.25 ± 0.01	2.27 ± 0.01
SFA (%)	38.11 ± 0.01 ^b^	44.4 ± 0.2 ^a^	30.2 ± 0.2 ^c^	26.37 ± 0.08 ^d^
MUFA (%)	17.1 ± 0.2 ^a^	9.06 ± 0.06 ^c^	7.0 ± 0.2 ^d^	10.9 ± 0.1 ^b^
PUFA (%)	44.8 ± 0.1 ^c^	46.5 ± 0.2 ^b^	62.8 ± 0.3 ^a^	62.77 ± 0.06 ^a^
**Tocopherols (mg/100 g fw)**				
α-Tocopherol	2.14 ± 0.05 ^d^	6.3 ± 0.1 ^c^	17.0 ± 0.2 ^a^	15.9 ± 0.4 ^b^
β-Tocopherol	6.8 ± 0.3 ^c^	5.8 ± 0.3 ^d^	13.8 ± 0.4 ^a^	12.7 ± 0.9 ^b^
γ-Tocopherol	21.2 ± 0.1 ^a^	9.5 ± 0.4 ^c^	12.4 ± 0.4 ^b^	8.4 ± 0.4 ^d^
δ-Tocopherol	nd	nd	10.2 ± 0.4	nd
Total tocopherols	30.1 ± 0.5 ^c^	21.7 ± 0.8 ^d^	53.4 ± 0.1 ^a^	37.0 ± 0.1 ^b^

nd—not detected. SFA—saturated fatty acids; MUFA—monounsaturated fatty acids; PUFA—polyunsaturated fatty acids. Statistically significant differences (*p* < 0.05) between samples were assessed by a one-way ANOVA, using Tukey’s significant difference (HSD) and are indicated by different letters.

**Table 3:** Retention time (Rt), wavelengths of maximum absorption (λmax), mass spectral data, and tentative identification of the phenolic compounds present in the hydroethanolic and aqueous extracts of *C. erecta*.

Peak	Rt (min)	λmax (nm)	[M-H]^−^ (*m/z*)	MS^2^ (*m/z*)	Tentative Identification
**1**	7.19	324	593	503 (31), 473 (10), 431 (100), 311 (14)	Apigenin-7-*O*-hexosyl-8-*C*-hexoside
**2**	8.74	272/327	593	533 (10), 503 (61), 473 (100), 413 (11), 383 (25), 353 (32)	Apigenin-6,8-di-*C*-hexoside
**3**	9.24	271/345	579	561 (11), 519 (19), 489 (23), 459 (100), 399 (31), 369 (15)	Luteolin-6-*C*-hexosyl-8-*C*-pentoside derivative I
**4**	10.19	270/343	579	561 (12), 519 (13), 489 (45), 459 (100), 399 (39), 369 (11)	Luteolin-6-*C*-hexosyl-8-*C*-pentoside derivative II
**5**	12.15	272/331	563	503 (12), 443 (100), 383 (23), 353 (32)	Apigenin 6-*C*-hexosyl-8-*C*-pentoside
**6**	13.19	272/327	563	503 (71), 443 (100), 383 (34), 353 (63)	Apigenin 6-*C*-pentosyl-8-*C*-hexoside
**7**	14.18	271/343	549	531 (18), 489 (32), 459 (100), 441 (23), 429 (13), 399 (23), 369 (22)	Luteolin-6-*C*-pentosyl-8-*C*-pentoside derivative I
**8**	15.89	271/342	549	531 (23), 489 (34), 459 (100), 441 (18), 429 (5), 399 (35), 369 (36)	Luteolin-6-*C*-pentosyl-8-*C*-pentoside derivative II
**9**	16.16	272/328	533	515 (52), 473 (71), 443 (100), 383 (28), 353 (25)	Apigenin-6,8-di-*C*-pentoside
**10**	17.09	335	431	311 (30), 283 (100)	Hydroxy-Puerarin
**11**	17.96	327	463	301 (100)	Quercetin-3-*O*-hexoside
**12**	19.21	320	549	505 (10), 463 (32), 301 (100)	Quercetin-*O*-malonyl-hexoside
**13**	21.19	325	447	301 (100)	Quercetin-*O*-deoxyhexoside

The identification of phenolic compounds was performed based on their chromatographic behaviour, UV-vis and mass spectra by comparison with standard compounds, when available. Otherwise, identification was attempted based on data reported in the literature.

**Table 4:** Quantification (mg/g extract) of the phenolic compounds-glycosylated and *O*-glycosylated flavonoids present in the hydroethanolic and aqueous extracts of *C. erecta* (mean ± SD, *n* = 3).

Peak	Flowering Stem		Stem after Flowering		Flowering Leaf		Leaf after Flowering	
	Hydroethanolic	Aqueous	Hydroethanolic	Aqueous	Hydroethanolic	Aqueous	Hydroethanolic	Aqueous
1	nd	nd	nd	nd	nd	0.334 ± 0.07	nd	nd
2	0.396 ± 0.002 ^a^	nd	0.09 ± 0.002 ^f^	0.22 ± 0.004 ^d^	0.284 ± 0.003 ^c^	0.33 ± 0.03 ^b^	0.32 ± 0.03 ^b^	0.171 ± 0.004 ^e^
3	tr	nd	tr	nd	tr	nd	nd	nd
4	0.384 ± 0.009	tr	nd	tr	tr	nd	nd	tr
5	2.96 ± 0.06 ^a^	0.39 ± 0.02 ^b^	nd	0.235 ± 0.004 ^d^	0.258 ± 0.001 ^c^	0.39 ± 0.02 ^b^	0.184 ± 0.004 ^e^	0.088 ± 0.001 ^f^
6	1.485 ± 0.005 ^a^	0.124 ± 0.005 ^f^	nd	0.258 ± 0.01 ^c^	0.21 ± 0.003 ^d^	0.29 ± 0.03 ^b^	nd	nd
7	3.27 ± 0.01	tr	nd	tr	nd	tr	nd	tr
8	0.47 ± 0.03	tr	nd	tr	nd	nd	tr	tr
9	0.891 ± 0.009 ^a^	0.17 ± 0.003 ^c^	nd	nd	0.349 ± 0.001 ^b^	nd	nd	nd
10	nd	nd	nd	nd	nd	0.77 ± 0.04	nd	nd
11	0.237 ± 0.015	nd	nd	nd	nd	nd	nd	nd
12	0.064 ± 0.001	nd	nd	nd	nd	nd	nd	nd
13	0.104 ± 0.009	nd	nd	tr	tr	nd	tr	tr
**TFC**	10.26 ± 0.01 ^a^	0.679 ± 0.02 ^e^	0.09 ± 0.002 ^h^	0.71 ± 0.02 ^d^	1.103 ± 0.003 ^c^	2.1 ± 0.1 ^b^	0.51 ± 0.02 ^f^	0.259 ± 0.005 ^g^
**TCGF**	9.849 ± 0.005 ^a^	0.679 ± 0.02 ^e^	0.09 ± 0.002 ^h^	0.71 ± 0.02 ^d^	1.102 ± 0.003 ^c^	2.1 ± 0.1 ^b^	0.51 ± 0.02 ^f^	0.259 ± 0.005 ^g^
**TOGF**	0.406 ± 0.005	nd	nd	tr	tr	nd	tr	tr

nd—not detected; tr—traces (> limit of detection (LOD) and <limit of quantification (LOQ)); TFC—Total Flavonoid Compounds; TCFG—Total *C*-Glycosylated Flavonoids; TOFG—Total *O*-Glycosylated Flavonoids. Standard calibration curves used for quantification: apigenin-6-*C*-glucoside (*y* = 197,337*x* + 30,036, *R^2^* = 0.9997, LOD = 0.19 µg/mL; LOQ = 0.63 µg/mL, peaks 1, 2, 5, 6, and 9), daidzin (*y* = 27,652*x* + 29187, *R^2^* = 0.9996, LOD = 20.58 µg/mL; LOQ = 62.35 µg/mL, peak 10), and luteolin-6-*C*-glucoside (*y* = 4087.1*x* + 72,589, *R^2^* = 0.999, LOD = 0.86 μg/mL; LOQ = 1.67 μg/mL, peaks 3, 4, 7, and 8). Statistically significant differences (*p* < 0.05) between samples were assessed by a one-way ANOVA using Tukey’s significant difference (HSD) and are indicated by different letters.

**Table 5:** Antioxidant and anti-inflammatory activities of the hydroethanolic and aqueous extracts of *C. erecta* (mean ± SD, *n = 3*).

			Flowering Stem	Stem after Flowering	Flowering Leaf	Leaf after Flowering
TBARS inhibition (EC_50_; µg/mL) ^a^		Aqueous	74.8 ± 0.7 ^a^	18.4 ± 0.2 ^c^	63.4 ± 0.9 ^b^	63 ± 1 ^b^
		Hydroethanolic	75.1 ± 0.7 ^c^	638 ± 8 ^b^	1060 ± 78 ^a^	63 ± 2 ^d^
OxHLIA (EC_50_; µg/mL)	∆*t* = 60 min	Aqueous	97 ± 6 ^b^	115 ± 7 ^a^	53 ± 3 ^d^	56 ± 3 ^c^
		Hydroethanolic	80 ± 3 ^c^	103 ± 2 ^a^	89 ± 4 ^b^	58 ± 3 ^d^
	∆*t* = 120 min	Aqueous	392 ± 11 ^a^	353 ± 6 ^b^	211 ± 9 ^c^	139 ± 6 ^d^
		Hydroethanolic	170 ± 9 ^c^	189 ± 2 ^b^	248 ± 5 ^a^	113 ± 5 ^d^
Anti-inflammatory activity (EC_50_ μg/mL)		Aqueous	>400	>400	>400	>400
		Hydroethanolic	157 ± 7	>400	>400	>400

Statistically significant differences (*p* < 0.05) between samples were assessed by a one-way ANOVA using Tukey’s significant difference (HSD) and are indicated by different letters. Antioxidant activity was expressed as EC_50_: extract concentration corresponding to a 50% of antioxidant activity. Trolox EC_50_ values: 5.4 ± 0.1 µg/mL (TBARS inhibition), 21.8 ± 0.3 µg/mL (OxHLIA Δ*t* = 60 min) and 44 ± 1 µg/mL (OxHLIA Δ*t* = 120 min.). Dexamethasone EC_50_ values: 6.3 ± 0.4 µg/mL (Anti-inflammatory activity).

**Table 6:** Antimicrobial and antifungal activity of the hydroethanolic and aqueous extracts of *C. erecta* (mean ± SD, *n = 3*).

Antibacterial Activity (mg/mL)							
		Gram-Positive Bacteria			Gram-Negative Bacteria		
		*S. aureus*	*B. cereus*	*L. monocytogenes*	*E. coli*	*S.* Typhimurium	*E. cloacae*
		MIC/MBC	MIC/MBC	MIC/MBC	MIC/MBC	MIC/MBC	MIC/MBC
**Flowering stem**	Aqueous	0.5/1	1/2	1/2	1/2	2/4	2/4
	Hydroethanolic	1/2	0.5/1	1/2	0.5/1	1/2	1/2
**Stem after flowering**	Aqueous	1/2	1/2	1/2	1/2	1/2	1/2
	Hydroethanolic	1/2	0.5/1	1/2	0.5/1	1/2	1/2
**Flowering leaf**	Aqueous	1/2	1/2	1/2	1/2	1/2	1/2
	Hydroethanolic	0.5/1	0.25/0.5	0.5/1	0.5/1	1/2	0.5/1
**Leaf after flowering**	Aqueous	1/2	0.5/1	1/2	1/2	1/2	1/2
	Hydroethanolic	1/2	0.5/1	1/2	1/2	1/2	1/2
**E211**		4/4	0.5/0.5	1/2	1/2	1/2	2/4
**E224**		1/1	2/4	0.5/1	0.5/1	1/1	0.5/0.5
**Antifungal activity (mg/mL)**							
		***A. fumigatus***	***A. niger***	***A. ochraceus***	***P. funiculosum***	***P. verrucosum* var. *cyclopium***	***T. viride***
		**MIC/MFC**	**MIC/MFC**	**MIC/MFC**	**MIC/MFC**	**MIC/MFC**	**MIC/MFC**
**Flowering stem**	Aqueous	0.5/1	0.5/1	0.5/1	0.5/1	1/2	1/2
	Hydroethanolic	0.5/1	1/2	0.5/1	0.5/1	0.5/1	1/2
**Stem after flowering**	Aqueous	0.25/0.5	0.5/1	0.5/1	0.5/1	1/2	1/2
	Hydroethanolic	0.5/1	1/2	0.5/1	0.5/1	0.5/1	0.5/1
**Flowering leaf**	Aqueous	0.5/1	0.5/1	0.5/1	0.5/1	0.5/1	0.5/1
	Hydroethanolic	0.5/1	0.5/1	0.5/1	0.5/1	0.5/1	0.5/1
**Leaf after flowering**	Aqueous	0.5/1	0.5/1	0.5/1	0.5/1	1/2	1/2
	Hydroethanolic	1/1	1/2	0.5/1	0.5/1	0.5/1	0.5/1
**E211**		1/2	1/2	1/2	1/2	2/4	1.0/2.0
**E224**		1/1	1/1	1/1	0.5/0.5	1/1	0.5/0.5

## Supplementary Materials

The following supporting information can be downloaded at: https://www.mdpi.com/article/10.3390/foods12010192/s1, Table S1: Proximal composition, moisture and energy value of frequently consumed WEPs in Brazil.
