# Supplementary material for: Chemical Composition and Biological Activity of Commelina erecta: An Edible Wild Plant Consumed in Brazil
Source: Foods. 2023 Jan 1;12(1):192. doi: 10.3390/foods12010192 (PMC9818490; doi:10.3390/foods12010192)
Supplement: Supplementary file 1 [file foods-12-00192-s001.zip › foods-2095266-supplementary.pdf]

Article

Chemical Composition and Biological Activity of *Commelina erecta*: An Edible Wild Plant Consumed in Brazil

Lucas Vinicius Cavichi [1,2,3], Ângela Liberal [1,2], Maria Inês Dias [1,2], Filipa Mandim [1,2], José Pinela [1,2], Marina Kostić [4], Marina Soković [4], Daneysa Lahis Kalschne [3], Ângela Fernandes [1,2,*], Cristiane Canan [3], Lillian Barros [1,2]
and Joana S. Amaral [1,2]

[1] Centro de Investigação de Montanha (CIMO), Instituto Politécnico de Bragança, Campus de Santa Apolónia, 5300-253 Bragança, Portugal; lucascavichi@hotmail.com (L.V.C.); angela.liberal@ipb.pt (Â.L.); maria.ines@ipb.pt (M.I.D.); filipamandim@ipb.pt (F.M.); jpinela@ipb.pt (J.P.); lillian@ipb.pt (L.B.); jamaral@ipb.pt (J.S.A.)

[2] Laboratório Associado para a Sustentabilidade e Tecnologia em Regiões de Montanha (SusTEC), Instituto Politécnico de Bragança, 5300-253 Bragança, Portugal

[3] Department of Food Sciences, Federal Technological University of Paraná, Medianeira 85884-000, Brazil;
daneysa@hotmail.com (D.L.K.); cristianecanan@gmail.com (C.C.)

[4] Institute for Biological Research "Siniša Stanković"-National Institute of Republic of Serbia, University of Belgrade, 11000 Belgrade, Serbia; marina.kostic@ibiss.bg.ac.rs (M.K.); mris@ibiss.bg.ac.rs (M.S.)

* Correspondence: afeitor@ipb.pt

Table S1. Proximal composition, moisture and energy value of frequently consumed WEPs in Brazil (data adapted from references [35-37]).

	Moisture (g/ 100 g fw [1])	Lipids (g/ 100 g fw)	Protein (g/ 100 g fw)	Carbohydrates (g/ 100 g fw)	Energy (kcal/100g fw)	Reference
Eruca vesicaria ssp (Arugula)	91.71	0.66	2.58	3.65	25.00	[36]
Pereskia aculeata (Ora-pro-nóbis)	88.09	0.30	3.10	5.01	35.14	[37]
Portulaca oleracea (Purslane)	92.86	2.03	3.39	0.36	20.00	[36]
Spilanthes oleracea L. (Jambu)	88.60	0.25	3.08	5.30	35.86	[35]

[1] fw – fresh weigh.

References:

35. Aguiar, J.P.L.; Yuyama, L.K.O.; Souza, F. das C. do A.; Pessoa, A. Biodisponibilidade do ferro do jambu (*Spilanthes oleracea* L.): estudo em murinos. *Rev. Pan-Amazônica Saúde* **2014**, *5*, 19–24.
36. Duarte, J.M.; Areco, K.C.N. Tabela de Composição Química Dos Alimentos. http://tabnut.dis.epm.br/Credito (accessed on November 14, 2022)
37. Queiroz, C.R.A. dos A..; Melo, C.M.T..; Andrade, R.R.; Pavani, L.C..; Morais, S. a. L. *34a Reunião Anual da Sociedade Brasileira de Química*. Instituto Federal do Triângulo Mineiro (IFTM) Campus Uberlândia, Brazil, 2009.
